# The impact of production of extended-spectrum β-lactamases on the 28-day mortality rate of patients with *Proteus mirabilis* bacteremia in Korea

**DOI:** 10.1186/s12879-017-2431-8

**Published:** 2017-05-03

**Authors:** Jin Young Ahn, Hea Won Ann, Yongduk Jeon, Mi Young Ahn, Dong Hyun Oh, Yong Chan Kim, Eun Jin Kim, Je Eun Song, In Young Jung, Moo Hyun Kim, Wooyoung Jeong, Nam Su Ku, Su Jin Jeong, Jun Yong Choi, Dongeun Yong, Young Goo Song, June Myung Kim

**Affiliations:** 1 Department of Internal Medicine, Seoul Medical Center, Seoul, South Korea; 2 Department of Internal Medicine, Yonsei University College of Medicine, Seoul, South Korea; 3 AIDS Research Institute, Yonsei University College of Medicine, Seoul, South Korea; 4 Department of Internal Medicine, Inje University College of Medicine, Ilsan Paik Hospital, Goyang, South Korea; 5 Department of Laboratory Medicine, Yonsei University College of Medicine, Seoul, South Korea; 6 Department of Internal Medicine and AIDS Research Institute, Yonsei University College of Medicine, 50-1 Yonsei-ro, Seodaemun-gu, Seoul, 120-752 South Korea

**Keywords:** *Proteus Mirabilis*, Bacteremia, Extended-spectrum β-lactamase, Mortality

## Abstract

**Background:**

The incidence of *Proteus mirabilis* antimicrobial resistance, especially that mediated by extended-spectrum β-lactamases (ESBLs), has increased. We investigated the impact of ESBL production on the mortality of patients with *P. mirabilis* bacteremia in Korea.

**Methods:**

Patients diagnosed with *P. mirabilis* bacteremia between November 2005 and December 2013 at a 2000-bed tertiary care center in South Korea were included in this study. Phenotypic and molecular analyses were performed to assess ESBL expression. Characteristics and treatment outcomes were investigated among ESBL-producing and non-ESBL-producing *P. mirabilis* bacteremia groups. A multivariate analysis of 28-day mortality rates was performed to evaluate the independent impact of ESBLs.

**Results:**

Among 62 *P. mirabilis* isolates from 62 patients, 14 expressed ESBLs (CTX-M, 2; TEM, 5; both, 6; other, 1), and the 28-day mortality rate of the 62 patients was 17.74%. No clinical factor was significantly associated with ESBL production. The 28-day mortality rate in the ESBL-producing group was significantly higher than that in the non-ESBL-producing group (50% vs. 8.3%, *p* = 0.001). A multivariate analysis showed that ESBL production (odds ratio [OR], 11.53, 95% confidence interval [CI], 2.11–63.05, *p* = 0.005) was independently associated with the 28-day mortality rate in patients with *P. mirabilis* bacteremia.

**Conclusions:**

ESBL production is significantly associated with mortality in patients with bacteremia caused by *P. mirabilis*. Rapid detection of ESBL expression and prompt appropriate antimicrobial therapy are required to reduce mortality caused by *P. mirabilis* bacteremia.

## Background

*Proteus mirabilis* is not a common cause of bloodstream infections in normal hosts, accounting for 1–3% of all episodes of bacteremia. [1–3] However, *P. mirabilis* is an important causative pathogen of various community- and healthcare-associated infections, such as wound infections, primary bacteremia, pneumonia and urinary tract infections, particularly among patients with anatomical or functional urinary tract abnormalities or indwelling urinary catheters [4–6].

The incidence of antimicrobial resistance of *P. mirabilis* has increased, and this can negatively affect prognosis. [7] The prevalence of multidrug-resistant (MDR) strains producing extended-spectrum β-lactamases (ESBLs), AmpC β-lactamases or carbapenemases has increased worldwide. [8–13] Among MDR isolates, ESBL-producing strains are the most frequent. [5, 9, 14] Previous studies reported that infections caused by MDR *P. mirabilis* strains are associated with higher rates of antibiotic treatment failure and mortality. [5, 8, 14] However, the treatment outcomes of MDR and non-MDR *P. mirabilis* infections are reported to be similar in several reports. [9, 15] The impact of MDR in *P. mirabilis* infections on treatment outcomes is thus unclear.

In Korea, the clinical aspects of *P. mirabilis* bacteremia have not been assessed, although microbiological data and molecular analyses of ESBLs from *P. mirabilis* strains have been reported. [16–18] Therefore, further study of the effects of MDR on clinical outcomes is needed. To this end, we investigated the clinical characteristics and antimicrobial susceptibility profile of *P. mirabilis* bacteremia isolates in Korea. We also evaluated the treatment outcomes of patients with ESBL-producing *P. mirabilis* bacteremia, in particular the impact of ESBL expression on the mortality rate of patients with *P. mirabilis* bacteremia.

## Methods

### Study design and patients

A retrospective cohort study was conducted at a 2000-bed, tertiary-care medical center in Seoul, South Korea. Microbiology laboratory databases were searched to identify all blood cultures positive for *P. mirabilis* among hospitalized patients older than 18 years from November 2005 to December 2013. Among the identified patients, only those with stored *P. mirabilis* isolate samples were included in the study, and their medical records were reviewed. For patients with more than one episode of *P. mirabilis* bacteremia, only data relevant to the first episode were analyzed. Isolates were divided into two groups according to ESBL production (the ESBL-producing and non-ESBL-producing groups). Microbiological and clinical factors were compared between the two groups to evaluate the factors and outcomes associated with ESBL production in *P. mirabilis* bacteremia. The study was approved by the Institutional Review Board (IRB) of Yonsei University Health System Clinical Trial Center. Since the study was retrospective, and the data of the subjects were anonymized, the IRB waived the requirement for written informed consent from the patients.

### Microbiological tests and molecular detection of ESBL

*P. mirabilis* was identified using either the ATB 32 GN or VITEK 2 system (bioMérieux, Marcy-L’Étoile, France). Antimicrobial susceptibility was determined using the disk-diffusion method or VITEK-2 N131 card (bioMérieux, Hazelwood, MO, USA). The results were interpreted according to the Clinical and Laboratory Standards Institute (CLSI) 2014 guidelines. [19] *P. mirabilis* isolates were stored in skim milk at −70 °C until further examination. In our institution, until 2010, ESBL production was assessed using the double-disk potentiation test. However, from 2011, ESBL production was no longer reported, in accordance with the revised CLSI 2011 guidelines. [20] Thus, we retrospectively performed the double-disk potentiation test to identify ESBL-production. Briefly, each isolate was subcultured twice from the skim milk prior to being tested. All 62 *P. mirabilis* isolates were inoculated onto the plates of Muller-Hilton agar (Becton Dickinson, Cockeysville, Md.) using ceftazidime and cefotaxime disks with and without clavulanic acid. The interpretation was followed by CLSI 2014 guidelines. [19] Genes encoding ESBLs (*bla*_TEM_, *bla*_SHV_ and *bla*_CTX-M_) were detected by polymerase chain reaction amplification using previously reported primers and reaction conditions [21–23].

### Collected data and definitions

The data collected included age, sex, date of culture, possible source of bacteremia, underlying diseases, various predisposing factors, laboratory data at the time of bacteremia diagnosis, severity of disease calculated by the sequential organ failure (SOFA) score and acute physiological and chronic health evaluation (APACHE) II score, treatment outcomes, antimicrobial therapy regimen and results of antimicrobial susceptibility testing.

Bacteremia was defined as the isolation of *P. mirabilis* from at least one separately obtained blood culture with clinical symptoms and signs compatible with infection. [24] Comorbidities were defined according to the International Classification of Disease, 10th Revision. [25] Predisposing conditions were taken into consideration only if they occurred within 1 month before the bacteremia. Previous exposure to specific antibiotics was considered in the analysis only if the antibiotics had been administered for at least 3 consecutive days within 1 month before the bacteremia.

After performing blood cultures, patients were promptly treated with empirical antibiotics within 24 h according to their predicted focus of infection until the susceptibility profiles of the isolates had been determined. The appropriateness of the prescribed antimicrobial therapy was evaluated retrospectively at the time of the bacteremia episode. Antimicrobial therapy was considered inappropriate if the isolated *P. mirabilis* did not show susceptibility to all administered antibiotics in vitro. Death within 28 days after bacteremia was regarded as being associated with *P. mirabilis* bacteremia unless definite clinical data suggested another cause of death.

### Statistical analysis

All statistical analyses were performed using the SPSS software, version 21 (SPSS; IBM Corp., Armonk, NY, USA) Continuous variables are presented as means ± [standard deviation (SD)] or medians [interquartile range (IQR)], and categorical variables are presented as numbers and percentages. To compare the two groups, the Student’s *t*-test or Mann-Whitney *U*-test, depending on the validity of the normality assumption, was used for continuous variables. The chi-squared test or Fisher’s exact test was used to assess categorical variables. Multivariate analysis was performed using logistic regression to identify factors that independently and significantly affected outcomes. Variables with a *p* value <0.05 in the univariate analysis were considered for inclusion in a multivariate model, and the final variables for inclusion in the multivariate model were selected via the backward likelihood ratio test. Survival analysis was performed using the Kaplan-Meier method, and comparison between the two groups was performed by log-rank test. Values of *p* < 0.05 were considered to indicate significance.

## Results

### Prevalence of ESBL production in *P. mirabilis* bacteremia

From November 2005 to December 2013, a total of 85 patients with *P. mirabilis* bacteremia were identified: 5 in 2005/2006, 7 in 2007, 9 in 2008, 8 in 2009, 10 in 2010, 18 in 2011, 17 in 2012 and 11 patients in 2013. Among them, 62 patients with stored bacterial strain samples were included in the analysis. Fourteen of 62 patients harbored ESBL-producing *P. mirabilis* strains (prevalence, 22.6%).

### Antimicrobial susceptibility of *P. mirabilis*

**Fig. 1:**
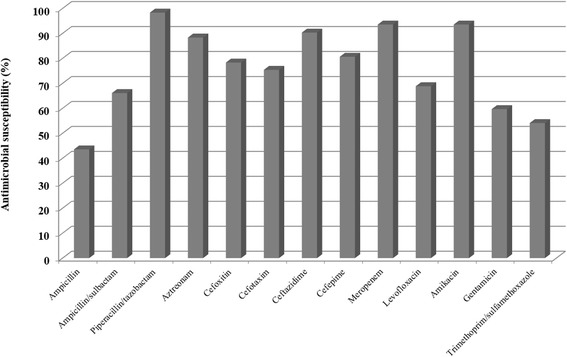
In vitro antimicrobial susceptibility tests for *Proteus mirabilis* isolates causing bacteremia

*The* in vitro antimicrobial susceptibilities of the *P. mirabilis* isolates are shown in Fig. 1. Most isolates were susceptible to meropenem (93.5%), piperacillin/tazobactam (98.3%), amikacin (93.5%), ceftazidime (90.3%), azteronam (88.3%) and cefepime (80.6%). The non-ESBL-producing group showed higher rates of susceptibility to most β-lactam antibiotics than did the ESBL-producing group; however, there was no significant difference in the rate of susceptibility to piperacillin/tazobactam between the two groups. Also, there were not significant differences in the rates of susceptibility to levofloxacin and trimethoprim for both groups; in contrast, the non-ESBL-producing group exhibited a higher rate of susceptibility to aminoglycosides (Table 1).

**Table 1:** In vitro antimicrobial susceptibility of ESBL producing and ESBL non-producing *P. mirabilis* isolates

Antimicrobial agent	No. (%) of susceptible isolate		*p* value
	non-ESBL-producing group *N* = 48	ESBL producing group*N* = 14	
Ampicillin	26 (54.2)	1 (7.1)	0.002
Ampicillin/sulbactam	37 (77.1)	4 (28.6)	0.003
Piperacillin/tazobactam	47 (97.9)	12 (100)	0.617
Aztreonam	45 (95.7)	8 (61.5)	0.004
Cefoxitin	42 (87.5)	5 (41.7)	0.002
Cefotaxim	43 (89.6)	3 (23.1)	<0.001
Ceftazidime	46 (95.8)	10 (71.4)	0.02
Cefepime	46 (95.8)	4 (28.6)	<0.001
Meropenem	44 (91.7)	14 (100)	0.297
Levofloxacin	36 (75.0)	6 (46.2)	0.088
Amikacin	47 (97.9)	11 (78.6)	0.033
Gentamicin	34 (70.8)	3 (21.4)	0.001
Trimethoprim /sulfamethoxazole	29 (60.4)	4 (30.8)	0.057^+^

ESBL, extended spectrum β-lactamases

### Clinical characteristics and mechanisms of ESBL production in patients with *P. mirabilis* bacteremia

The baseline characteristics of the patients are shown in Table 2. The median age was 74.5 years in the ESBL-producing group and 71 years in the non-ESBL-producing group. Patients in the ESBL-producing and non-ESBL-producing groups were predominantly male and female, respectively; however, this was not a significant difference. Urinary tract infection was the most frequent source of infection in the non-ESBL-producing group, while a significantly higher frequency of pneumonia, as a source of bacteremia, was seen in the ESBL-producing group (42.9% vs. 6.3%, *p* = 0.003).

**Table 2:** Baseline characteristics and clinical outcomes of patients with *P. mirabilis* bacteremia

Factors	non-ESBL-producing group *N* = 48	ESBL producing group *N* = 14	*p* value
Age, y, median(IQR)	71.0 (60.5–79)	74.5 (61.0–80.25)	0.706
Age ≥ 65 years, n(%)	30 (60.0)	9 (64.3)	0.903
Male, n(%)	18 (37.5)	9 (64.3)	0.075
BMI, kg/m^2^,median(IQR)	22.37 (20.51–25.54)	21.21 (18.80–23.46)	0.247
Infection source			
Urinary tract infection, n(%)	28 (58.3)	4 (28.6)	0.05
Pneumonia, n(%)	3 (6.3)	6 (42.9)	0.003
Skin and soft tissue infection, n(%)	1 (2.1)	1 (7.1)	0.403
Biliary infection, n(%)	9 (18.8)	0 (0)	0.105
Catheter related infection, n(%)	2 (4.2)	1 (7.1)	0.543
Others, n(%)	5 (10.5)	2 (14.3)	0.61
Comorbidities			
HTN, n(%)	31 (64.6)	7 (50)	0.324
DM, n(%)	19 (39.6)	5 (35.7)	0.794
Cardiovascular disease, n(%)	4 (8.3)	4 (28.6)	0.069
Chronic kidney disease, n(%)	6 (12.5)	1 (7.1)	1.000
Chronic liver disease, n(%)	3 (6.3)	3 (21.4)	0.122
Solid tumor, n(%)	25 (52.1)	7 (50)	0.891
Hematologic malignancy, n(%)	2 (4.2)	0 (0)	1.000
Solid organ transplantation, n(%)	3 (6.3)	0 (0)	1.000
Charlson score, median(IQR)	2.0 (1.0–2.75)	2.0 (1.0–3.25)	0.151
Predisposing factors			
Neutropenia, n(%)	2 (4.2)	0 (0)	1.000
Chemotherapy, n(%)	5 (10.4)	3 (21.4)	0.365
Nursing home residence, n(%)	7 (14.6)	0 (0)	0.334
Hemodialysis, n(%)	7 (14.6)	4 (28.6)	0.249
ICU care, n(%)	11 (22.9)	8 (57.1)	0.022
Maintaining foley catheter, n(%)	11 (22.9)	6 (42.9)	0.141
Maintaining PEG tube, n(%)	1 (2.1)	4 (28.6)	0.008
Previous antibiotic use	13 (27.1)	7 (50)	0.12
Cephalosporins, n(%)	6 (12.5)	2 (14.3)	1.000
Carbapenems, n(%)	2 (4.2)	2 (4.2)	0.217
Fluorquinolones, n(%)	2 (4.2)	1 (7.1)	0.543
BLBLIs, n(%)	4 (8.4)	1 (7.1)	1.000
Clinical presentation			
Shock, n(%)	17 (35.4)	6 (42.9)	0.612
Acute kidney injury, n(%)	15 (31.3)	2 (14.3)	0.313
APACHE II score, median(IQR)	12.0 (8.0–16.75)	13.5 (10.75–19.25)	0.115
SOFA score, median(IQR)	3.0 (1.0–5.75)	4.0 (1.0–6.5)	0.85
Outcomes			
Inappropriate antimicrobial therapy, n(%)	9 (18.8)	6 (42.9)	0.082
Overall mortality, n(%)	25 (52.1)	11 (78.6)	0.077
28-day mortality, n(%)	4 (8.3)	7 (50)	0.001

*ESBL* extended spectrum β-lactamases, *IQR* interquartile range, *BMI* body mass index, *HTN* hypertension, *DM* diabetes mellitus, *ICU* intensive care unit, *PEG* percutaneous endoscopic gastrostomy, *BLBLIs* beta-lactam/beta-lactamase inhibitors, *APACHE II score* Acute Physiology and Chronic Health Evaluation II score, *SOFA score* the Sequential Organ Failure Assessment score

Among the ESBL-producing strains, two produced TEM only, five produced CTX-M only, and six produced both TEM and CTX-M. One isolate showed a positive reaction in a double-disk potentiation test but did not express TEM-, CTX-M- or SHV-type enzymes.

### Factors associated with ESBL production by *P. mirabilis*

A univariate analysis was conducted to investigate the factors associated with bacteremia caused by ESBL-producing *P. mirabilis* (Table 2). There were no significant differences in the rates of underlying comorbidities or most of the predisposing factors between the ESBL-producing and non-ESBL-producing groups. However, the rates of receiving intensive care unit (ICU) treatment and maintaining a percutaneous endoscopic gastrostomy (PEG) tube were significantly higher in the ESBL-producing group than in the non-ESBL-producing group (57.1% vs. 22.9%, *p* = 0.022 for ICU, 28.6% vs. 2.1%, *p* = 0.008 for PEG tube). Initial clinical presentations and severity scores did not show meaningful differences between the two groups. However, in the multivariate analysis, no variable that was significant in the univariate analysis was independently associated with ESBL production (Table 3).

**Table 3:** Multivariate analysis for associated factors of bacteremia caused by ESBL producing *P. mirabilis*

Factors	OR	95% CI	*p* value
Age	1.03	0.97–1.09	0.294
Female sex	0.51	0.11–2.29	0.375
Having pneumonia as source of infection	3.26	0.45–23.52	0.241
Maintaining PEG	9.96	0.64–153.98	0.101
Previous ICU care	3.15	0.69–14.29	0.137

*ESBL* extended spectrum β-lactamases, *PEG* percutaneous endoscopic gastrostomy, *ICU* intensive care unit, *OR* odd ratio, *CI* confidence interval.

### Treatment outcomes of *P. mirabilis* bacteremia

**Fig. 2:**
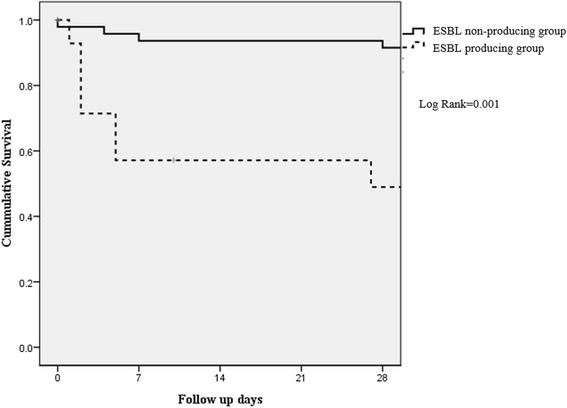
Kaplan-Meier survival estimates among patients with *P. mirabilis* bacteremia. ESBL, extended spectrum β-lactam

Treatment outcomes are also summarized in Table 2. Inappropriate antibiotics were prescribed more frequently on the day of bacteremia diagnosis in the ESBL-producing group (42.9% vs. 18.8%, *p* = 0.082), but the difference was not statistically significant. During the study period, 36 of 62 patients died, yielding an overall crude mortality rate of 58.6%. Among these 36 patients, 11 died within 28 days of the *P. mirabilis* bacteremia episode, yielding a 28-day mortality rate of 17.74%. The ESBL-producing group showed a higher 28-day mortality rate than that of the non-ESBL-producing group (50% vs. 8.3%, *p* = 0.001); this was also confirmed by a survival curve analysis (*p* = 0.001) (Fig. 2).

### Factors associated with 28-day mortality in patients with *P. mirabilis* bacteremia

Univariate and multivariate analyses of 51 survivors and 11 non-survivors were performed to identify the factors associated with 28-day mortality. As shown in Table 4, ESBL production by *P. mirabilis*, previous antibiotic use, and a higher baseline APACHE II score or SOFA score were significantly associated with 28-day mortality in patients with *P. mirabilis* bacteremia in the univariate analyses. In the multivariate analysis (Table 5), ESBL production (odds ratio [OR], 11.53; 95% confidence interval [CI], 2.11–63.05, *p* = 0.005) and a high SOFA score (OR 1.32, 95% CI 1.03–1.70, *p* = 0.029) were independently associated with 28-day mortality in patients with *P. mirabilis* bacteremia.

**Table 4:** Factors associated with 28-day mortality in patients with *P. mirabilis* bacteremia

Factors	Survivors *N* = 51 (%)	Nonsurvivors *N* = 11 (%)	*p* value
Age, y, median(IQR)	71.0 (63.0–79.0)	73.0 (54.0–84.0)	0.797
Age ≥ 65 years, n(%)	32 (62.7)	7 (63.6)	1.000
Sex, male, n(%)	21 (41.2)	6 (54.6)	0.51
BMI, kg/m^2^, median(IQR)	22.35 (10.07–25.52)	21.78 (19.15–23.29)	0.366
ESBL producing pathogen, yes, n(%)	7 (13.7)	7 (63.6)	0.001
Infection source			
Urinary tract infection, n(%)	28 (54.9)	4 (36.4)	0.264
Pneumonia, n(%)	6 (11.8)	3 (27.3)	0.191
Skin and soft tissue infection, n(%)	2 (3.9)	0 (0)	1.000
Biliary infection, n(%)	8 (15.7)	1 (9.1)	1.000
Catheter related infection, n(%)	2 (3.9)	1 (9.1)	0.449
Comorbities			
HTN, n(%)	33 (64.7)	5 (45.5)	0.311
DM, n(%)	21 (41.2)	3 (27.3)	0.505
Cardiovascular disease, n(%)	5 (9.8)	3 (27.3)	0.142
Chronic kidney disease, n(%)	7 (13.7)	0 (0)	0.334
Chronic liver disease, n(%)	3 (5.9)	3 (27.3)	0.063
Rheumatologic disease, n(%)	2 (3.9)	0 (0)	1.000
Solid tumor, n(%)	26 (51.0)	6 (54.5)	0.83
Hematologic malignancy, n(%)	1 (2.0)	1 (9.1)	0.326
Solid organ transplantation, n(%)	2 (3.9)	1 (9.1)	0.449
Charlson score, median(IQR)	2.0 (1.0–2.0)	2.0 (1.0–4.0)	0.239
Predisposing factors			
Neutropenia, n(%)	2 (4.0)	0 (0)	1.000
Chemotherapy, n(%)	7 (13.7)	1 (9.1)	1.000
Nursing home residence, n(%)	7 (13.7)	0 (0)	0.334
Hemodialysis, n(%)	8 (15.7)	3 (27.3)	0.394
Maintaining foley catheter, n(%)	11 (21.6)	6 (54.5)	0.056
Maintaining PEG tube, n(%)	4 (7.8)	1 (9.1)	1.000
ICU care, n(%)	13 (25.5)	6 (54.5)	0.077
Previous antibiotic use, n(%)	13 (25.5)	7 (63.6)	0.029
Cephalosporins, n(%)	6 (11.8)	2 (18.2)	0.623
Carbapenems, n(%)	3 (5.9)	1 (9.1)	0.552
Fluorquinolones, n(%)	1 (2.0)	2 (18.2)	0.079
BLBLI, n(%)	3 (5.9)	2 (18.2)	0.212
Clinical presentation			
Shock, n(%)	19 (37.3)	4 (36.4)	1.000
Acute kidney injury, n(%)	14 (27.5)	3 (27.3)	1.000
APACHE II score, median(IQR)	11.0 (8.0–16.0)	17.0 (11.0–19.0)	0.027
SOFA score, median(IQR)	2.0 (1.0–5.0)	5.0 (4.0–8.0)	0.033
Inappropriate antimicrobial therapy, n(%)	10 (19.6)	5 (45.5)	0.115

*IQR* interquartile range, *BMI* body mass index, *ESBL* extended spectrum β-lactamases, *HTN* hypertension, *DM* diabetes mellitus, *PEG* percutaneous endoscopic gastrostomy, *ICU* intensive care unit, *BLBLIs* beta-lactam/beta-lactamase inhibitors, *APACHE II score* Acute Physiology and Chronic Health Evaluation II score, *SOFA score* the Sequential Organ Failure Assessment score

**Table 5:** Multivariate analysis of risk factors for 28-day mortality

Factors	OR	95% CI	*p* value
ESBL producing	11.53	2.11–63.05	0.005
Previous antibiotics use	5.09	0.94–27.54	0.059
SOFA score	1.32	1.03–1.69	0.029

*ESBL* extended spectrum β-lactamases, *SOFA score* the Sequential Organ Failure Assessment score, *OR* odd ratio, *CI* confidence interval

## Discussions

Infections caused by Enterobacteriaceae expressing ESBLs are a healthcare concern worldwide; however, studies of ESBL production have focused on *Klebsiella pneumoniae* and *Escherichia coli.* [26–31] *P. mirabilis* is an important emerging pathogen, particularly in healthcare settings, due to its potential for horizontal transmission and drug resistance. [32] Reports on the antimicrobial resistance of *P. mirabilis* have increased in recent years. [32–35].

Four previous clinical studies on bacteremia caused by MDR *P. mirabilis* strains have addressed the risk factors for acquisition of antimicrobial resistance and the impact of MDR on mortality. [5, 8, 14, 15] Two of these studies focused on ESBL-producing strains [8, 15], while the other two evaluated MDR strains defined as non-susceptible to at least one agent in three or more classes of antimicrobials. [5, 14] The risk factors for acquiring resistant *P. mirabilis* strains were receiving nursing home care [5, 8, 14], previous antibiotic treatment [5, 14, 15], hemodialysis [15], recurrent hospitalization [5, 14], urinary catheterization [5, 8] and having a peptic ulcer or peripheral vascular disease [14]. Only one of the four studies reported no differences in recurrence or mortality rates between MDR and non-MDR strains [15]; the others reported significantly higher mortality rates in patients with MDR *P. mirabilis* bacteremia. [5, 8, 14] In this study, no clinical factor was significantly associated with ESBL production and the 28-day mortality rate in the ESBL-producing group was significantly higher than that in the non-ESBL-producing group.

The prevalence of ESBLs in *P. mirabilis* varies among studies from 0.7% to 57% [15, 36–42]; however, many studies reported that the prevalence has increased over time. [36, 38–40] Previous studies from 2005 to 2011 in Korea reported incidences of ESBL-producing *P. mirabilis* of 6.5–12.6%, but no information regarding the change in incidence over time was provided. [16, 18, 43] In this study, the prevalence of ESBL production among *P. mirabilis* bacteremia isolates was 22.6% over an 8-year period, which is higher than that reported by previous studies in Korea.

Most ESBLs are CTX-M-, TEM- and SHV-type β-lactamases. [31, 44] Recently, CTX-M-type β-lactamases have become the predominant type in many areas. [37, 38, 41, 45, 46] The distribution of ESBL type varies geographically; TEM-type enzymes are the most common ESBLs in some areas. [8, 40, 47, 48] According to one Korean study of ESBL-producing *P. mirabilis* isolates in 2005, most ESBL producers possessed the *bla*_TEM_ gene, followed by bla_CTX-M_. [16] In this study, of the 14 isolates from the ESBL-producing group, 78.5% (11/14) produced CTX-M, 50% (7/14) produced TEM, and 49% (6/14) produced both CTX-M and TEM; CTX-M type and TEM-type enzymes were also predominant in this study.

The *P. mirabilis* isolates showed a high rate of susceptibility to meropenem. Only 4 of 62 strains were not susceptible to meropenem, all of which were in the non-ESBL-producing group. Among them, one strain isolated in 2012 showed intermediate susceptibility to meropenem minimum inhibitory concentration (MIC) 2 mg/L but was susceptible to piperacillin/tazobactam and other third- and fourth-generation cephalosporins. The other three strains (two strains with meropenem disk zone diameters of 21 mm and one strain with meropenem MIC of 4 mg/L) were isolated between 2008 and 2009 and were deemed susceptible prior to the retrospective adjustment according to the modified CLSI guidelines. [19] The carbapenem-resistant isolate (one strain with meropenem MIC of 4 mg/L) showed decreased susceptibility to ampicillin, ampicillin/sulbactam, aminoglycosides, quinolones and trimethoprim/sulfamethoxazole but was susceptible to piperacillin/tazobactam and other third- and fourth-generation cephalosporins.

Almost all *P. mirabilis* isolates were susceptible to piperacillin/tazobactam, irrespective of ESBL production. Piperacillin/tazobactam is less effective than meropenem against bacteremia caused by ESBL-producing Enterobacteriaceae [49–51]. Therefore, piperacillin/tazobactam can be used empirically prior to determination of the susceptibility profile of potential or predicted ESBL-producing *P. mirabilis* isolates.

In this study, the ESBL-producing group showed a significantly higher 28-day mortality rate than that of the non-ESBL-producing group. The prognosis of bacteremia caused by Enterobacteriaceae is associated with several factors, such as the clinical severity at the time of antimicrobial treatment, underlying diseases and the appropriateness of antibiotics. [52] The higher rate of inappropriate initial antimicrobial treatment in patients with ESBL-producing *P. mirabilis* could explain the increased mortality rate. [5, 26, 53, 54] However, in this study, clinical severity, underlying diseases and the rate of receiving inappropriate initial antibiotics did not differ between the ESBL-producing and non-ESBL-producing groups. Although the analyses were not limited to *P. mirabilis* infection, worse clinical outcomes from infections caused by ESBL-producing pathogens susceptible to the antibiotics used have been reported. [52, 55, 56] Kim et al. reported that the favorable treatment response rate was significantly lower in the ESBL group than in the non-ESBL group among 68 patients with bacteremia caused by *E. coli* or *K. pneumoniae* treated with extended-spectrum cephalosporins, to which the infecting organisms were susceptible in vitro. [52] The mean interval from the time of bacteremia to the administration of presumptively appropriate antimicrobial agents was not different between the two groups. [52] Although the research did not make use of the revised cephalosporin breakpoints, the authors concluded that ESBL production itself resulted in a worse prognosis of bacteremia. [52] Use of certain extended-spectrum cephalosporins for infections caused by ESBL-producing pathogens can result in treatment failure. In particular, a favorable response to treatment with a third-generation cephalosporin other than ceftazidime has been reported in some cases of infection with ESBL-producing strains. [27] Moreover, the molecular type of ESBL can also affect the treatment outcomes. [27, 57, 58] Finally, an inoculum effect, i.e., a significant increase in the MIC with an increasing number of organisms, has been suggested to explain the worse prognosis associated with infections caused by ESBL-producing pathogens treated with antibiotics other than carbapenems [55].

Infections with ESBL-producing strains can be treated early with appropriate antibiotics before the availability of antimicrobial susceptibility profiles in the presence of clinical risk factors predictive of infection with ESBL-producing strains. However, no clinical factor was significantly associated with ESBL production in this study. Therefore, rapid detection of ESBL expression is essential for early appropriate treatment. Microbiologic identification using matrix-assisted laser desorption/ionization time-of-flight mass spectrometry and rapid susceptibility testing facilitates faster detection of pathogens and prompt prescription of appropriate antibiotics, which may lead to improved clinical outcomes [59–61].

This study was subject to several limitations. First, the type of CTX-M and TEM β-lactamases was not identified, and ESBL-producing strains were not subjected to pulsed-field gel electrophoresis assay. Therefore, the ESBL types and role of horizontal spread of their encoding genes could not be assessed. Next, stored *P. mirabilis* isolate samples were available for only 64 of 85 patients infected with *P. mirabilis* bacteremia during the study period. Therefore, we were unable to evaluate the incidence of ESBL-producing isolates over time. Other limitations include the retrospective nature and small sample size of the study. Nevertheless, our findings suggest that ESBL production exerts a negative influence on the mortality rate of patients with *P. mirabilis* bacteremia.

## Conclusions

In conclusion, our results suggest that ESBL production is significantly associated with the 28-day mortality rate, in that patients with bacteremia caused by ESBL-producing *P. mirabilis* have higher 28-day mortality rates. Because no clinical factor was found to be predictive of ESBL production by *P. mirabilis* bacteremia isolates, early detection of ESBL expression and prompt appropriate antimicrobial therapy are essential for improving the prognosis.

## Abbreviations

APACHE: acute physiological and chronic health evaluation
CI: confidence interval
CLSI: Clinical and Laboratory Standards Institute
ESBL: extended-spectrum β-lactamase
ICU: intensive care unit
IQR: interquartile range
IRB: Institutional Review Board
MDR: multidrug-resistant
MIC: minimum inhibitory concentration
OR: odds ratio
PEG: percutaneous endoscopic gastrostomy
SD: standard deviation
SOFA: sequential organ failure
